# The Effect of Composition of Different Ecotoxicological Test Media on Free and Bioavailable Copper from CuSO_4_ and CuO Nanoparticles: Comparative Evidence from a Cu-Selective Electrode and a Cu-Biosensor

**DOI:** 10.3390/s111110502

**Published:** 2011-11-03

**Authors:** Aleksandr Käkinen, Olesja Bondarenko, Angela Ivask, Anne Kahru

**Affiliations:** 1 Laboratory of Molecular Genetics, National Institute of Chemical Physics and Biophysics, Akadeemia tee 23, Tallinn 12618, Estonia; E-Mails: aleksandr.kakinen@kbfi.ee (A.K.); olesja.bondarenko@kbfi.ee (O.B.); 2 Department of Chemical and Materials Technology, Tallinn University of Technology, Ehitajate tee 5, Tallinn 19086, Estonia; 3 Department of Gene Technology, Tallinn University of Technology, Ehitajate tee 5, Tallinn 19086, Estonia

**Keywords:** copper-containing nanoparticles, bacteria, bioluminescent bioreporter, ion-selective electrode, speciation, toxicity, complexation

## Abstract

The analysis of (bio)available copper in complex environmental settings, including biological test media, is a challenging task. In this study, we demonstrated the potential of a recombinant *Pseudomonas fluorescens*-based biosensor for bioavailability analysis of CuSO_4_ and CuO nanoparticles (nano-CuO) in seventeen different ecotoxicological and microbiologial test media. In parallel, free Cu in these test media was analysed using Cu-ion selective electrode (Cu-ISE). In the case of CuSO_4_, both free and bioavailable Cu decreased greatly with increasing concentration of organics and phosphates in the tested media. A good correlation between free and bioavailable Cu was observed (r = 0.854, p < 0.01) indicating that the free Cu content in biological test media may be a reasonably good predictor for the toxicity of CuSO_4_. As a proof, it was demonstrated that when eleven EC_50_ values for CuSO_4_ from different organisms in different test media were normalized for the free Cu in these media, the difference in these EC_50_ values was decreased from 4 to 1.8 orders of magnitude. Thus, toxicity of CuSO_4_ to these organisms was attributed to the properties of the test media rather than to inherent differences in sensitivity between the test organisms. Differently from CuSO_4_, the amount of free and bioavailable Cu in nano-CuO spiked media was not significantly correlated with the concentration of organics in the test media. Thus, the speciation of nano-CuO in toxicological test systems was not only determined by the complexation of Cu ions but also by differential dissolution of nano-CuO in different test conditions leading to a new speciation equilibrium. In addition, a substantial fraction of nano-CuO that was not detectable by Cu-ISE (*i.e*., not present as free Cu-ions) was bioavailable to Cu-biosensor bacteria. Thus, in environmental hazard analysis of (nano) particulate materials, biosensor analysis may be more informative than other analytical techniques. Our results demonstrate that bacterial Cu-biosensors either in combination with other analytical/speciation techniques or on their own, may serve as a rapid (eco)toxicological screening method.

## 1. Introduction

Copper is a microelement necessary for various vital functions, but at the same time one of the most toxic heavy metals for aquatic organisms (e.g., crustaceans, algae, bacteria [1]), acting adversely already at sub-ppm concentrations. Indeed, soluble copper salts have been extensively used as pesticides. During the past decade, copper-containing nanoparticles are also increasingly appearing in various applications, elevating the risk of their environmental release upon usage or disposal of the respective products.

It is widely accepted that bioavailability and subsequently, the toxic effects of heavy metals, depend on their speciation. Thus, metal speciation in different environmental matrices (natural waters, soils, sediments) has received remarkable attention [2] and the corresponding information is considered crucial for proper (environmental) risk analysis. Although metal speciation is usually the main concern in environmental matrices, every standard laboratory toxicological assay needs to be performed in certain specific conditions, e.g., test media and temperature, which may influence the metal speciation and consequently, the test results [3]. In toxicity testing, the test medium has to support e.g., viability, growth or reproduction of the test organisms. Although for most of the standardized toxicity assays the media used are described by OECD, ISO, ASTM norms, it may vary depending on the test organism and type of the test. However, for an (eco)toxicological test to result in environmentally relevant and accurate prediction of metal toxicity, the estimation of metal complexing potential of the test media is of vital importance [4]. Currently, the theme of differential heavy metal complexing potential of toxicological test media has received a new impulse in the context of rapidly increasing nano(eco)toxicological data and their interpretation. It has been suggested that the vast knowledge and experience obtained from metal toxicity and speciation studies may also be applicable to metal-containing nanomaterials. This is mainly derived from emerging experimental data which indicate that dissolution of metal-containing nanomaterials may be one of the main issues underlying their toxicity [5].

Despite the importance of heavy metal speciation in interpretation of the results of toxicological assays, the available techniques to determine metal speciation and bioavailability are relatively limited. One of the most robust and probably the most widely applied techniques for speciation analysis is the ion-selective electrode (ISE) [6,7]. Unfortunately, ISEs that have the capacity of detecting free ions only exist for limited types of metal ions [7]. Furthermore, although extremely high sensitivity of some electrodes has been reported (up to 10^−11^ M [8]), the detection limits of ISEs are generally too high (e.g., in case of Cu-ISE usually 10^−6^ M, *i.e*., 63.5 μg Cu/L) to be applied for environmental samples. In addition, interference of ISEs with metal-organics complexes and non-target inorganic ions (Cl, Br, Fe, *etc*.) has been discussed [9,10]. Despite all these drawbacks, so far no other speciation technique has been able to outcompete ISEs. Often, the practical metal speciation measurements by ISEs have been accompanied by theoretical speciation modeling, most often performed using the Visual MINTEQ equilibrium model [11]. The results from chemical equilibrium models may be complemented with involve information about competitive binding of metals to organic ligands on biological surfaces in more complex models like Free Ion Activity Model or Biological Ligand Model and used to describe metal bioavailability [12]. On the other hand, there has been a considerable effort in developing simple bioassays that may be used for direct assessment of heavy metal bioavailability. An example of such bioassays are microbial biosensors [13,14], which allow specific detection of bioavailable metals through a highly sensitive biorecognition process followed by induction of a measurable signal, e.g., bioluminescence [15]. These metal-specific microbial biosensors have been applied for the analysis of bioavailable metals in soils, sediments, [16,17] and metal-containing nanomaterials [18,19].

In this study, we performed a comprehensive comparison between free and bioavailable copper (applied as CuSO_4_ and nano-CuO) determined using a Cu ion-selective electrode (Cu-ISE) and a Cu-specific bacterial biosensor. Seventeen different standard ecotoxicological and microbiological test media were analysed with the aim to: (i) determine the complexing potential of these media for CuSO_4_ and nano-CuO and (ii) compare the responses of the Cu-biosensor and Cu-ISE. In parallel, Cu speciation was calculated using the Visual MINTEQ equilibrium model. The results for Cu speciation in the selected test media were used to calculate the amount of free ions at reported experimentally determined toxicity values (E(I)C_50_) for different aquatic test organisms.

## 2. Experimental Section

### 2.1. Test Chemicals and Their Preparation for the Analysis with the Cu-ISE and Bacterial Cu-Biosensor

CuSO_4_·5H_2_O (analytical grade) was purchased from Riedel-de-Haën. 63.5 g Cu/L stock solution was prepared in deionised (DI) water and stored in the dark at room temperature. CuSO_4_·5H_2_O was added to the test media as 100-fold concentrated stock solution in water.

Nano-CuO (advertised particle size 30 nm) was purchased from Sigma-Aldrich. The primary size of nano-CuO was confirmed to be 31 ±12.8 nm in an earlier study by Blinova *et al*. [20]. TEM and SEM images of nano-CuO preparation are shown elsewhere [20,21]. 63.5 g Cu/L stock suspension on nano-CuO was made in DI water; the suspension was sonicated for 30 min as described earlier [18]. Hydrodynamic diameter of nano-CuO in DI water (measured from 20 mg/L suspension using Zetasizer Nano-ZS; Malvern Instruments, UK) was 195 ± 2 nm. Nano-CuO was added to the test media as 100-fold concentrated stock suspension in water. Hydrodynamic size of nano-CuO in final media was measured as in case of DI water; average hydrodynamic diameter and Pdi (polydispersity index) were calculated from three parallel measurements.

### 2.2. Test Media

The list of standard ecotoxicological and microbiological media used in this study is presented in Table 1. All mineral salts used for preparation of the media were of analytical grade. Cas-aminoacids (AA) (casein hydrolysate), Tryptone, Yeast extract and Malt extract were from LabM (Lancashire, UK), Peptone was from Difco Laboratories (Beckton Dickinson, MD, USA). The test media were prepared by dissolving the desired amount of ingredients in DI water, autoclaved (121 °C for 15 min) or filter-sterilized (0.1 μm filter pore size, Minisart) and stored at room temperature.

Concentration of Cu in the test media was also determined by AAS-graphite furnace method according to the standard procedures (EVS-EN ISO/IEC 17025:2005) in a certified laboratory of the Institute of Chemistry, Tallinn University of Technology (Estonia). Additionally, pH (Orion PerpHect ROSS) and conductivity (EcoScan CON 5 conductometer; Eutech Instruments, Singapore) of the test media were measured. Conductivity of DI water was measured to be 0.0003 mS/cm.

### 2.3. Analysis of Free Cu Using a Cu Ion-Selective Electrode

A copper ion-selective electrode 96–29 ionplus (Orion Research, Thermo Scientific, MA, USA) was used. Before measurement, the electrode was thoroughly washed with DI water, then with 0.025 M H_2_SO_4_, followed by polishing of the sensor surface with Al_2_O_3_ polishing strip. Electrode was calibrated daily using 10^−8^ M (6.4 × 10^−4^ mg Cu/L)–10^−1^ M (6,350 mg Cu/L) CuSO_4_·5H_2_O dilutions in DI water. *Prior* measurement, the ionic strength of all solutions was adjusted by supplementing the sample with 0.1 M NaNO_3_. Ionic strength of the test solution was adjusted because of its importance in the response of ISE as shown by Sauvé *et al*. [22]. Appropriate dilutions of CuSO_4_ and nano-CuO were prepared in 5 mL of test media and the measurements were conducted in 30 mL polypropylene tubes. Dilutions of nano-CuO were allowed equilibrate for 2 h at 30 °C before the measurement. The limit of detection (LOD) of Cu-ISE in each test media was calculated as recommended for ion-selective electrodes by IUPAC [23]. Briefly, log(10) of the added Cu was plotted against the electrode potential and the crossing point between the linear increase of the electrode potential and the line representing the electrode background potential was designated as the limit of detection (Cu-ISE_LOD_) (Figure S1). The LODs and respective standard deviations were calculated from three independent measurements. Free Cu was calculated using the following equation:

(1) $$\text{Free}\;\text{Cu}(\%)=\frac{\text{Cu}\;{\text{ISE}}_{\text{LOD}}\;\text{in}\;\text{test}\;\text{media}}{\text{Cu}\;{\text{ISE}}_{\text{LOD}}\;\text{in}\;\text{DI}\;\text{water}}\times 100$$

In addition, the Cu-ISE results were used to calculate free Cu at EC_50_ of different organisms for CuSO_4_ and nano-CuO. The EC_50_ values were obtained from earlier published studies (except that of *S. cerevisiae* in YPD media that was personal communication from Dr. K. Kasemets, NICPB, Estonia). Experimental EC_50_ values were obtained in exactly the same media (composition of the media was verified) that were used for Cu speciation analysis in this study (Table 1). To calculate the amount of free Cu at EC_50_ values for CuSO_4_ or nano-CuO in different media, the potential of Cu-ISE at EC_50_ concentrations in these media was measured and compared to CuSO_4_ or nano-CuO concentrations that induced similar electrode potential in DI water (Figure S2). The respective concentration in DI water was considered as the concentration of free Cu at this EC_50_ value.

### 2.4. Calculation of Free Ion Concentration Using Visual MINTEQ

Chemical equilibrium model Visual MINTEQ 2.51 [28] was used to calculate Cu ion speciation in CuSO_4_ solutions prepared in different mineral media. Due to the lack of respective equilibrium models, no calculations were performed for complex organics-containing media. For mineral media, respective pH, concentrations of all main cations and anions were used as input; temperature was set to 23 °C. The sum of free and hydrated Cu ions was considered as the gross free Cu in the test media.

### 2.5. Analysis of Bioavailable Cu Using a Cu-Biosensor Bacterium

The Gram-negative Cu-sensing *Pseudomonas fluorescens* OS8::KnCueRPcopAlux, in which bioluminescence is specifically induced by bioavailable Cu ions [15], was used to measure bioavailable Cu. Sensor bacteria were pre-grown overnight on a shaker (200 rpm, 30 °C) in 3 mL of LB medium (Table 1) supplemented with 100 μg/L of kanamycin. 20 mL of fresh LB was inoculated with 1/50 diluted overnight culture, and bacteria were grown until mid-exponential phase (OD_600_ of 0.6), and cells were separated by centrifugation at 5,000 ×g for 10 min. Cell pellet was washed twice with 20 mL of appropriate test medium and further diluted with the same medium until OD_600_ ∼ 0.1 (approximately 10^6^ bacterial cells/mL). 100 μL of CuSO_4_ or nano-CuO dilution or medium only (blank medium control) was pipetted onto white polypropylene 96-well microplate (Greiner Bio-one, Germany); 100 μL of bacterial suspension was added to each well and plates were incubated at 30 °C for 2 h. Bioluminescence was measured using Orion II luminometer (Berthold Detection Systems, Germany) and response of sensor bacteria to copper compounds was calculated as follows:

(2) $$\text{Induction}\;(\text{fold})=\frac{\text{Biolumines}\;\text{cence}\;\text{in}\;\text{Cu}\;\text{containing}\;\text{sample}}{\text{Biolumines}\;\text{cence}\;\text{in}\;\text{blank}\;\text{medium}}$$

Two parallel experiments were included per individual assay and three independent individual assays were performed. Due to the different potency of the cells to induce bioluminescence in ‘nutritionally’ different media absolute induction values in these media differed. Thus, the induction of the bacterial biosensor was expressed as %, where maximum induction value was considered as 100 and induction in blank medium as 1. Limit of the detection (LOD) of the Cu-biosensors was set at 20% induction (Figure S3).

## 3. Results and Discussion

### 3.1. Comparative Response of the Cu Ion Selective Electrode (Cu-ISE) and the Bacterial Cu-Biosensor to CuSO_4_ in Standard Ecotoxicological and Microbiological Media

In this study, we analysed the speciation (free ion content) and bioavailability of copper in seventeen different media. The media selection included various standard ecotoxicological test media: 2% NaCl (used for Microtox toxicity test with bioluminescent bacteria *Vibrio fischeri*), algal medium (used for the toxicity testing with *Pseudokirchneriella subcapitata* according to OECD 201), two artificial freshwaters (used for tests with crustaceans *Daphnia magna* according to OECD 202 and with *Thamnocephalus platyurus*). In addition, common media used for cultivation of microorganisms were included: LB medium (an undefined rich medium that supports the growth of variety of bacteria), malt extract (ME) and YPD (Yeast Peptone Dextrose medium) both used for the cultivation of yeasts (Table 1). Also, M9 and HMM media that were supplemented with 0.5% Cas-amino acids (AA) were studied. The M9 medium has been used in our previous studies for toxicity evaluation of metals and organic chemicals to *Escherichia coli* [29] and for the analysis of bioavailable metals using metal-inducible bioluminescent bacterial sensors [15,30]. HMM medium has been specifically suggested for the analysis of heavy metals due to its minimal metal-complexing capability [27]. Also 0.9% NaCl and its Cas-amino acid (AA) supplemented versions were tested as 0.1% AA amended 0.9% NaCl has been applied by us earlier to study the bioavailability and toxicity of CuO nanomaterials [18,20,21]. Cu speciation and complexing potential of these different laboratory test media were studied using two methods: (i) a Cu-ISE that responds to free Cu ions and (ii) a Cu-specific bacterial biosensor that responds to bioavailable Cu.

#### 3.1.1. Response of Cu-ISE to CuSO_4_: Measurement of Free Cu

**Figure 1.**
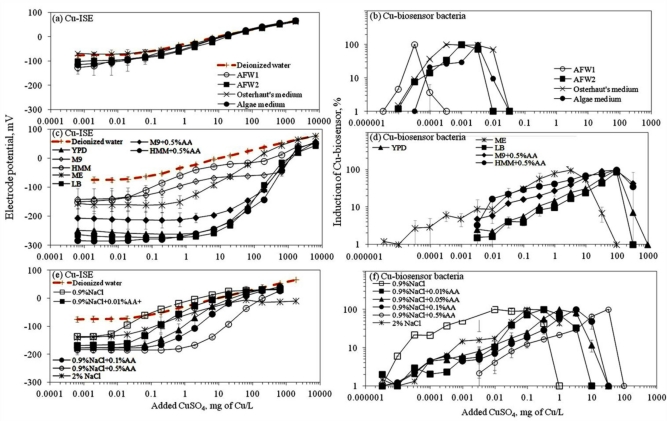
Response of Cu-ISE (left panels) and Cu-biosensor *Pseudomonas fluorescens* OS8::KnCueRPcopAlux (right panels) to CuSO_4_ in different ecotoxicological and microbiological media. (**a**,**b**) ‘poor’ mineral media; (**c**,**d**) organics-containing media (LB, ME, YPD) and mineral media (M9, HMM) supplemented with 0.5% of Cas-amino acids; (**e**,**f**) 0.9% NaCl with various concentrations of Cas-amino acids (AA). Numeric values of respective LODs are presented in Table 2.

Ion selective electrodes (ISEs) have been relatively widely applied to study the speciation of e.g., certain heavy metals in different environmental conditions [31] and the corresponding standard protocols have been developed [22]. Thus, speciation analysis using ISEs may be considered as a well established method. Response of ISEs has been considered to indicate the content of free ions of the studied elements and has been often correlated with bioavailability and toxicity of these elements [32,33]. Speciation analysis of CuSO_4_ in seventeen selected laboratory test media showed that most of the media contained ligands capable of complexing the Cu ions and thus, reduced the amount of free Cu (see calibration curves in Figure 1). As expected, the limit of detection of the Cu-ISE (Cu-ISE_LOD_) (Table 2) was lowest in test media with no or low organics content. Overall, the order of Cu-ISE_LOD_ for CuSO_4_ in different tested media was: 0.9% NaCl ≅ AFW1 < 2% NaCl < Osterhout’s medium ≅ HMM < AFW2 ≅ 0.9% NaCl + 0.01%AA < algal medium < M9 ≅ 0.9% NaCl + 0.05%AA < 0.9% NaCl+0.1%AA < malt extract < HMM + 0.5%AA < 0.9% NaCl + 0.5%AA < M9 + 0.5%AA < YPD < LB. Interestingly, in some mineral media—Osterhout’s medium, AFW1, HMM, 0.9% and 2% NaCl—the LOD of Cu-ISE was lower than that in DI water (considered to contain 100% free Cu ions) (Figure 1, Table 2). As discussed by Lanza [34], this may be due to the presence of interfering ions like Cl^−^ in these media. Indeed, the difference in electrode potential leading to abnormally low LOD values was observed only at low Cu concentrations; at higher Cu concentrations the difference between the electrode potential in DI water and these media disappeared (Figure 1(a)). However, the media showing very low Cu-ISE_LOD_ values—Osterhout’s medium, AFW1, HMM, 0.9% NaCl and 2% NaCl—contained the highest amount of free Cu ions also according to Visual MINTEQ equilibrium model (Figure S4). At the same time, the amount of free Cu was remarkably lower in other mineral media—algal medium, AFW2 and M9 mineral media—in which the prevalent Cu species were Cu-EDTA, CuCO_3_ and CuHPO_4_, respectively (Figure S4). The prevalence of these species could be expected. Indeed, EDTA (in algal medium) is a well-known trace metal chelator [35] and phosphates have been demonstrated to form strong metal-phosphate complexes that often precipitate [36]. Yet, phosphate-containing media like M9 or phosphate-buffered saline (PBS) are often used for toxicological tests as: (i) phosphates have a good buffering capacity and (ii) are required for several physiological functions of living cells. However, when phosphates were substituted with morpholinepropane sulfonic acid (MOPS) for buffering capacity and organic phosphate (β-glycerophosphate) to serve as physiological phosphate supply in HMM media as suggested by [37,38], the fraction of free Cu was significantly increased compared to phosphate-containing media (Table 2, Figure 1).

Response of Cu-ISE to CuSO_4_ in organics-containing media (Figure 1(c)) was generally in correlation with organics content of the media. This was clearly evident in the case of 0.9% NaCl where the gradual addition of AA was accompanied by a respective increase of the Cu-ISE_LOD_. Addition of 0.5% (wt) of AA to 0.9% NaCl increased the Cu-ISE_LOD_ by 87-fold (Table 2). Analogously, the addition of 0.5% AA to M9 and HMM mineral media increased the LOD of the Cu-ISE about 30-fold. Indeed, complexation of Cu by organic ligands and formation of relatively strong complexes in ‘rich’ media is a known phenomenon [36,39]. The following step in the current study was to compare the results from Cu-ISE with the response of bacterial Cu-biosensor in the same test media.

#### 3.1.2. Response of Bacterial Cu-Biosensor to CuSO_4_: Measurement of Bioavailable Cu

Contrary to Cu-ISE, which is a well established method for metal speciation analysis, bacterial metal-specific biosensors are currently largely in their developmental stage. Indeed, despite their more than 20-year history, they have not been widely applied yet for real environmental analysis [13]. Most likely, the development of these bacterial sensor cells has been inhibited by the ongoing dispute about the actual fraction of the metal that is triggering their biological/analytical response and thus, by difficulties in interpreting the obtained results. According to [40] the response of microbial biosensor cells may be exclusively due to the soluble ionic forms of metals. However, recently Brandt *et al.* reported that in addition to free Cu, Cu-DOM (dissolved organic matter) complexes were also bioavailable to Cu sensor bacteria [41]. Due to the current uncertainty in the interpretation of biosensor results for environmental risk assessment, this technique suffers also from the lack of comprehensive validation and standardization of the method. By parallel analysis of Cu speciation in similar samples, the current study aims to draw correlations between the response of bacterial Cu-biosensor and Cu-ISE.

Before discussing the response of the bacterial biosensor to CuSO_4_ in different media, one must note that being a live bacterial cell, this recombinant biosensor is not ‘operating’ at extremely low ionic strength solutions *(*e.g., DI water). The latter usually results in a relative poor bioluminescent response of sensor bacteria to copper if analysed in low organics-containing media: the bioluminescence in live bacteria consumes considerable amounts of cellular energy [42] and in extremely low nutrient conditions the energy level may be insufficient. Due to different levels of bioluminescence produced by the biosensor cells in different media, we present the induction of the Cu-biosensor bacteria as percentage of the maximal induction (see Section 2.5).

**Figure 2.**
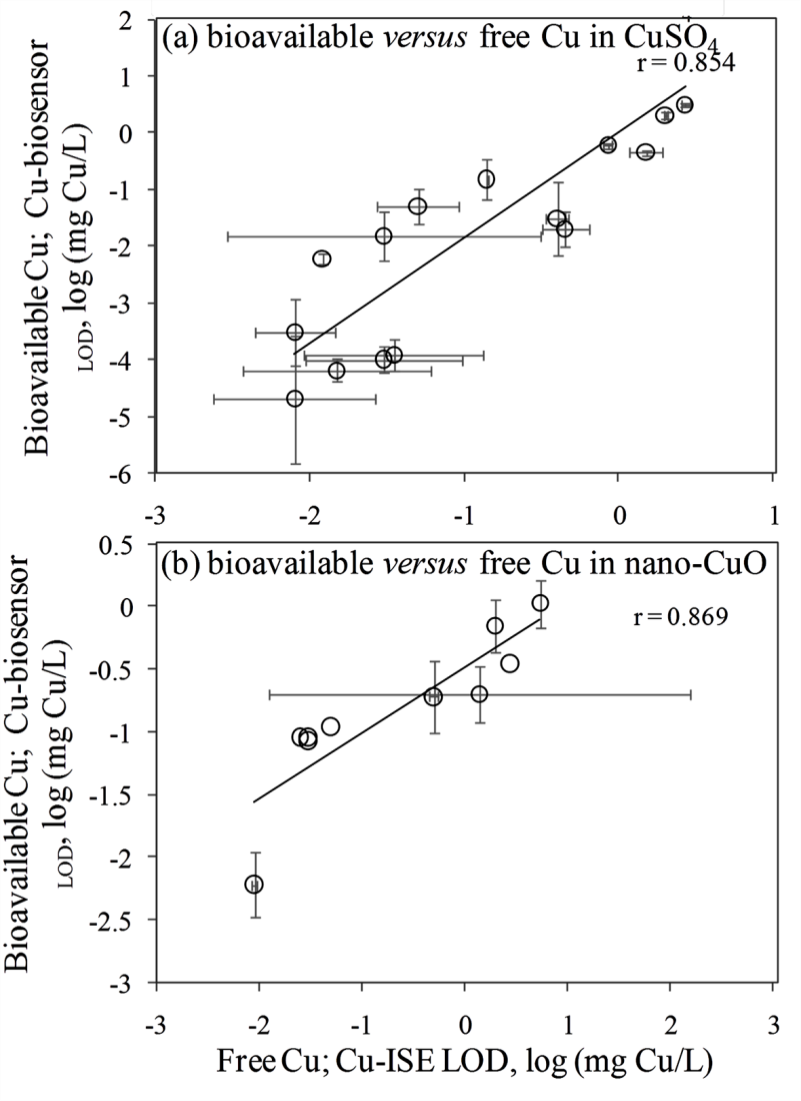
Correlation between limits of determination (LOD) of Cu-ISE (indicative of free Cu) and Cu-biosensor (indicative of bioavailable Cu) for CuSO_4_ in different ectotoxicological and microbiological media. Data are plotted from Tables 2 and 3. (**a**) Cu-ISE_LOD_ and Cu-biosensor_LOD_ for CuSO_4_; (**b**) Cu-ISE_LOD_ and Cu-biosensor_LOD_ for nano-CuO.

In general, the LOD values of the *P. fluorescens* OS8::KnCueRPcopAlux bacterial biosensor and Cu-ISE in CuSO_4_ spiked media were significantly correlated: the corresponding r value was 0.854 (Figure 2(a,b)) indicating that the bacterial sensor, at least in the media tested herein, responded mainly to free species of copper. Analogously to the Cu-ISE results, decrease of bioavailable Cu with increasing amount of organics in the media, was observed (see Cu-biosensor_LOD_ values in Table 2). The effect of media composition on Cu toxicity to bioluminescent bacterial cells and its relation with free Cu in these media has been demonstrated also by some other authors. For example, [33] showed that the bioluminescent response of bacteria was correlated with the free Cu in a set of soil pore waters. Also, [43] found a good correlation between free Cu measured by Cu-ISE and bacterial response in few samples analysed by them. Respectively, decrease in Cu toxicity with increasing dissolved organic carbon levels has also been shown by Apte *et al*. [44]. Our study showed that in addition to excellent correlation between free Cu ions and bioavailable Cu, the Cu-biosensor bacteria responded generally to remarkably lower CuSO_4_ levels than did Cu-ISE. Thus, bacterial biosensors used in the current study can be considered more sensitive warning systems for heavy metal-caused potential environmental hazard than the free metal ion measurement.

### 3.2. Comparative Response of Cu-Ion Selective Electrode and Bacterial Cu-Biosensor to Nano-CuO

There is a general belief that a fraction of toxicity of metal-containing nanomaterials may result from dissolved metal ions [45]. The release of Cu ions from CuO nanoparticles was reported as the main case of CuO toxicity for the crustaceans *Thamnocephalus platyurus*, the bacteria *Vibrio fischeri* [18] and *Escherichia coli* [21], the algae *Pseudokirchneriella subcapitata* [46] and the nematodes *Caenorhabditis elegans* [47]. Recently, Puzyn *et al*. [48] have developed a quantitative structure-activity relationship (QSAR) model for metal containing NPs using the formation of metal ions as a single predictor for their toxicity. Therefore, we decided to use a Cu-ISE and bacterial Cu-biosensor in parallel, to study the speciation of Cu in suspensions of CuO nanoparticles. Due to the poor bioluminescent response of Cu-biosensor bacteria in mineral media (Osterhout’s medium, AFW1, AFW2, algal medium, 2% NaCl and 0.9% NaCl; the induction of bioluminescence in these media was only about 10% of that in organics-containing media; data not shown) discussed above, we did not include these media in speciation analysis of nano-CuO.

**Figure 3.**
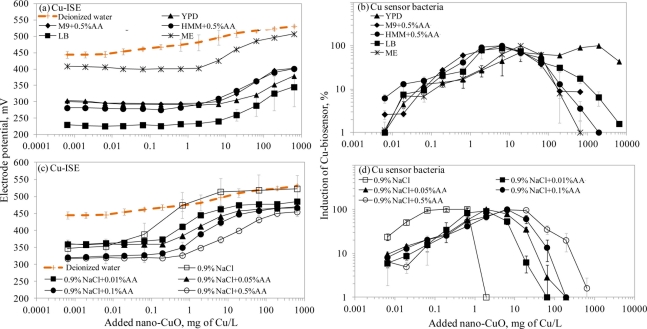
Response of Cu ion-selective electrode (Cu-ISE; left panels) and *Pseudomonas fluorescens* OS8::KnCueRPcopAlux Cu-biosensor (right panels) to nano-CuO in different ecotoxicological and microbiological media. (**a**,**b**) organics-containing media; (**c**,**d**) 0.9% NaCl with various concentrations of Cas-amino acids (AA).

**Figure 4.**
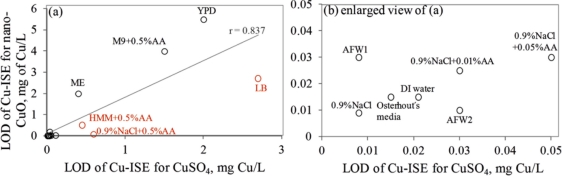
Correlation between Cu-ISE_LOD_ in CuSO_4_ and in nano-CuO suspensions prepared in different ecotoxicological and microbiological media. The names of test media correspond to Table 1; (**b**) is enlarged view of (**a**). Symbols in brown color indicate those media where the concentration of free ions in nano-CuO suspensions was lower than could be predicted from what was observed in case of CuSO_4_.

Interestingly, the limits of detection of Cu-ISE to CuSO_4_ (0.021 ± 0.005 mg Cu/L (Table 2)) and nano-CuO (0.015 ± 0.003 mg Cu/L (Table 3)) were almost identical. This indicates that at these low concentrations all Cu from nano-CuO was likely dissolved and present in the form of free ion. Analogously to CuSO_4_, the fraction of fee Cu ions decreased when nano-CuO was introduced to various laboratory test media (Figure 3). Although there was generally a good correlation between the free Cu ions in CuSO_4_ and in nano-CuO-spiked test media (r = 0.837; Figure 4) there were several exceptions. Specifically, in LB medium, HMM and 0.9% NaCl supplemented with 0.5%AA the concentration of free Cu in CuSO_4_ was not the best predictor for the free Cu concentration in suspensions of nano-CuO. Notably, more free Cu was detected in nano-CuO suspensions than could be predicted from the results of CuSO_4_ (Figure 4, the ‘outlier’-media are marked with brown color). Therefore, we suggest that differently from CuSO_4_, where the only process affecting the Cu speciation was interaction of Cu ions with media components, additional processes take place in case of nano-CuO. These processes likely include agglomeration of nanoparticles, release of Cu ions from the CuO, and finally, interaction of the released Cu ions with the media components. Unfortunately, dissolution and speciation of dissolved metals from metal-containing nanomaterials has not been studied in a systematic manner. Only a recent report by Gunawan *et al*. [49] demonstrated differential dissolution of CuO nanoparticles in different media. Their results showed that in organics-containing ‘rich’ media the dissolution of CuO was indeed high if compared to that in water or in saline solution. Similar observation has been done for nano ZnO in a study by Li *et al*. [50] where the authors suggested that the affinity of Zn for ligands present in these media was responsible for dissolution of ZnO. Organic ligand-enhanced dissolution due to the presence of proteins and organic substances in the test media has been observed also in other studies concerning CdSe, iron oxides, aluminium oxides and aluminium oxyhydroxides [51]. One reason for the enhanced dissolution may be the decrease in effective hydrodynamic size of the nanomaterials as a result from coating of the particles with organic molecules. This may lead to stabilization of the nanomaterials aggregates and increased dissolution. Thus, in our experiments differential dissolution of nano-CuO due to media components and differential speciation of dissolved Cu in the media is a very likely scenario. Behaviour of nano-CuO in 0.9% NaCl amended with different amounts of amino acids is a relevant example. Differently from CuSO_4_ in case of which the fraction of free Cu decreased with increasing amino acid content, very similar Cu-ISE_LOD_ as well as Cu-biosensor_LOD_ values for nano-CuO were obtained (Table 3). Thus, it could be supposed that with increasing amino acid content in the meedium, the dissolution of Cu from nano-Cu increased. In accordance with the hypothesis about organic ligand-induced decrease in effective hydrodynamic diameter of the nanomaterials, we observed that the Dh of nano-CuO decreased when Cas-aminoacids were added to saline (Table 3).

Overall, there was a good correlation between LODs of Cu-ISE and Cu-biosensor bacteria for nano-CuO (r = 0.869; Figure 2). However, in most of the tested media, the LOD of the Cu-biosensor was remarkably up to 5-fold lower than that of Cu-ISE (Figure 2; Table 3). This interesting observation suggests that in nano-CuO suspensions, bacterial cells were also able to access fractions of Cu other than just the free ion form detected by Cu-ISE. This finding is similar to our previous reports showing partial bioavailability of particulate matter bound Cu in soil and sediment samples [52]. Thus, we propose that in case of particle-containing samples (including Cu-containing nanomaterials), more Cu may be bioavailable to living (e.g., microbial) cells than could be predicted based on only dissolved fraction of Cu.

### 3.3. Toxicity of CuSO_4_ and Nano-CuO to Different (Eco)toxicological Model Organisms: Normalization to Free and Bioavailable Ions

Usually, the difference in toxicity of copper to several freshwater and saltwater organisms varies remarkably, ranging from 0.005 to 10 mg/L [53]. When a certain organism group is considered, the variation is smaller, but still considerable; in a recent review [5] acute toxicity data (48–96 h LC_50_) of Cu^2+^ for fish from the literature were compared. Thirteen EC_50_ values for zebrafish, rainbow trout, trout, common carp, gibel carp and mullet were included; these values ranged for almost two orders of magnitudes from 0.03 till 1.4 mg/L, the median value being 0.21 mg/L. According to [53], this variability may be due to the species differences but also due to differential speciation of copper in different test environments (media) used to perform these toxicity assays.

Here, we collected ‘in house’ toxicity data (EC_50_ values) for CuSO_4_ and nano-CuO which were determined using standard ecotoxicological test organisms (protozoa, crustaceans, algae, bacteria) and yeasts (Table 4). All these toxicity tests were performed in the same media that were used for Cu speciation and bioavailability analysis in the current study. The collected EC_50_ values of these organisms towards CuSO_4_ differed by 4 orders of magnitude (from 0.02 to 368 mg/L, Table 4). Using data from Cu-ISE, we calculated the amount of free Cu ions at each of the EC_50_ values. When we normalized the CuSO_4_ EC_50_ values for free Cu in the test media, the difference in EC_50_ values comprised just 1.8 orders of magnitude. Thus, in case of CuSO_4_, the free ion concentration may be considered as relatively suitable parameter for toxicity prediction, as also earlier demonstrated by Apte *et al*. [44]. To our surprise, a good agreement between the EC_50_ values and free Cu content was observed in our experiments, even though they were conducted with different organisms of substantially different biological complexity (ranging from bacteria to algae and crustaceans). This brings us to the conclusion that the apparently big differences observed in toxicity of CuSO_4_ towards these organisms could be attributed to the properties of the testing media rather than to inherent differences in sensitivity between the test organisms. Unfortunately, we were unable to calculate the bioavailable Cu corresponding to respective EC_50_ values using the Cu-biosensor bacteria as all the EC_50_ values for CuSO_4_ exhibited already strong toxic effects towards the Cu-biosensor (Figure 1). However, as the working principle of biosensor bacteria is to respond to subtoxic amounts of heavy metals, high toxicity of EC_50_ concentrations of Cu compounds to sensor bacteria was anticipated.

## 4. Conclusions

In this study, we used a Cu ion-selective electrode (Cu-ISE) and a bacterial Cu-biosensor in parallel, to analyze the speciation of CuSO_4_ and nano-CuO in seventeen selected laboratory test media. In case of CuSO_4_, both the ISE and sensor bacteria showed that organics-containing media contained lower free and bioavailable copper than did mineral media. However, in case of nano-CuO the ‘complexing’ effect was not so evident. It could be assumed that upon dispersion of nano-CuO in organics-containing media two simultaneous processes take place: (i) enhanced dissolution of copper due to the increased dispersion of CuO and (ii) complexing of dissolved Cu by organics present in the medium. Overall, the free Cu measured with the Cu-ISE and bioavailable Cu measured by the Cu-biosensor correlated well. Interestingly, in nano-CuO suspensions, more bioavailable Cu (bacterial sensor assay) than free Cu (ISE) was detected. Thus, we suggest that bacterial biosensors were able to access additional fraction of nano CuO that was not dissolved and detectable by Cu-ISE. Hence, in the environmental hazard analysis of metal-containing (nano) particulate materials, biosensor analysis may be more informative than other respective analytical techniques. We also demonstrated that the remarkable difference in sensitivity of various aquatic organisms towards copper may be largely explained by the differential speciation of this metal in the test media used. Therefore, although very different aquatic organisms were compared (bacteria, algae, yeasts, crustaceans, protozoa) the concentrations of free copper harmful to these organisms were quite similar. This suggests that there seems to be no big inherent differences in sensitivity towards copper between different types of organisms. Moreover, the effect of composition of the test media should be considered as one of the most important factors in interpreting the results of toxicity tests.

## Supplementary Information



## Figures and Tables

**Table 1.** Toxicological and microbiological test media used in this study.

**Designation of the media** (traditional test organism for this medium)	**Content*per*L**	**pH**	**Conductivity, mS/cm**	**Cu, mg/L^a^**	**Reference**
	***ECOTOXICOLOGICAL TEST MEDIA***				
**Osterhout’s medium** (protozoan *Tetrahymena* sp.)	104 mg NaCl, 8.5 mg MgCl_2,_ 4 mg MgSO_4_, 2.3 mg KCl, 1 mg CaCl_2_	5.2	0.29	<detection	[24]
**Artificial freshwater 1 (AFW1)** (crustacean *Daphnia* sp.*)*	294 mg CaCl_2_·2H_2_O, 123.25 mg MgSO_4_·7H_2_O, 64.75 mg NaHCO_3_, 5.75 mg KCl	7.8	0.64	<detection	OECD 202
**Artificial freshwater 2 (AFW2)** (crustacean *Thamnocephalus* sp.*)*	60 mg CaSO_4_·2H_2_O, 123 mg MgSO_4_·7H_2_O, 96 mg NaHCO_3,_ 4 mg KCl	7.8	0.24	<detection	[25]
**Algal medium** (algae *Pseudokirchneriella subcapitata*)	15 mg NH_4_Cl, 12 mg MgCl_2_·6H_2_O, 18 mg CaCl_2_·2H_2_O, 15 mg MgSO_4_·7H_2_O, 1.6 mg KH_2_PO_4_, 50 mg NaHCO_3_, 0.1 mg Na_2_EDTA·2H_2_O, 0.08 mg FeCl_3_·6H_2_O, 0.185 mg H_3_BO_3_, 0.415 mg MnCl_2_·4H_2_O, 3 × 10^−3^ mg ZnCl_2_, 1.5 × 10^−3^ mg CoCl_2_·6H_2_O, 7 × 10^−3^ mg Na_2_MoO_4_·2H_2_O, 10^−5^ mg CuCl_2_·2H_2_O	8.3	0.05	<detection	OECD 201
					
	***MICROBIOLOGICAL TEST MEDIA***				
**Malt extract (ME)** (yeasts *Saccharomyces cerevisiae)*	11 g maltose, 8 g carbohydrates, 1 g proteins	5.2	0.82	0.0076	Lab M, UK
**Yeast extract peptone dextrose (YPD)** (yeasts *Saccharomyces cerevisiae)*	20 g Bacto peptone, 10 g yeast extract, 20 g glucose	6.7	3.39	0.0089	[26]
**M9**^b^ (bacteria e.g., *Escherichia coli)*	6 g Na_2_HPO_4_, 3 g KH_2_PO_4_, 0.5 g NaCl, 1 g NH_4_Cl, 0.25 g MgSO_4_·7H_2_O, 0.01 g CaCl_2_	7.0	8.43	<detection	[26]

**M9 + 0.5%AA** (bacteria e.g., *Escherichia coli)*	6 g Na_2_HPO_4_, 3 g KH_2_PO_4_, 0.5 g NaCl, 1 g NH_4_Cl, 0.25 g MgSO_4_·7H_2_O, 0.01 g CaCl_2_, 5 g Cas-amino acids, 1 g glucose	7.0	9.92	0.0056	see previous
**LB** (bacteria e.g., *Escherichia coli)*	10 g tryptone, 5 g yeast extract, 5 g NaCl	7.0	19.55	0.0195	[26]
**Heavy metal MOPS medium (HMM)**^b^ (bacteria e.g., *Escherichia coli)*	8.4 g MOPS, 0.22 g glycerol-2-phosphate, 3.7 g KCl, 0.54 g NH_4_Cl, 0.06 g MgSO_4_, 0.162 mg FeCl_3_	7.2	8.9	<detection	[27]
**HMM + 0.5%AA** (bacteria e.g., *Escherichia coli)*	8.4 g MOPS, 0.22 g glycerol-2-phosphate, 3.7 g KCl, 0.54 g NH_4_Cl, 0.06 g MgSO_4_, 0.162 mg FeCl_3_, 5 g Cas-amino acids, 4 g glucose	7.2	8.9	0.0021	see previous
**2% NaCl** (marine bacteria e.g, *Vibrio fischeri)*	20 g NaCl	4.4	>20	n.a.	
					
**supplemented 0.9% NaCl**	***SUPPLEMENTED 0.9% SALINE***				
**0.9% NaCl**	9 g NaCl	5.8	16.6	n.a.	
**0.9% NaCl + 0.01%AA**	9 g NaCl, 1 g glucose, 0.1 g Cas-amino acids	6.1	16.8	n.a.	
**0.9% NaCl + 0.05%AA**	9 g NaCl, 1 g glucose, 0.5 g Cas-amino acids	6.3	17.0	n.a.	
**0.9% NaCl + 0.1%AA**	9 g NaCl, 1 g glucose, 1 g Cas-amino acids	6.2	17.2	0.0042	
**0.9% NaCl + 0.5%AA**	9 g NaCl, 1 g glucose, 5 g Cas-amino acids	6.2	18.8	0.0069	

a concentration of Cu in the test media measured by AAS (limit of determination 0.002 mg/L). All values <0.02 mg/L were designated as <detection;

b included to the analysis mineral controls for M9 + 0.5%AA and HMM + 0.5%AA; AA—amino acids;

n.a.—not analysed.

**Table 2.** Limit of detection (LOD) of the Cu-ion selective electrode (Cu-ISE) (indicative of free Cu ions) and *Pseudomonas fluorescens* OS8::KnCueRPcopAlux Cu-biosensor (indicative of bioavailable Cu) for CuSO_4_ in selected ecotoxicological and microbiological media. Average of three replicates ± standard deviation is shown. Data calculated from Figure 1.

**Designation of the medium^a^**	**Cu-ISE_LOD_, mg/L**	**Free Cu^2+^(mg/L) at Cu-ISE_LOD_^b^**	**Cu-biosensor_LOD_, mg/L**	**Free Cu^2+^(mg/L) at Cu-biosensor_LOD_^a^**
Deionized (DI) water ^c^	0.021 ± 0.005	0.021 ^c^	n.a	n.a.
Osterhout’s medium	0.015 ± 0.005	0.017	0.000065 ± 0.000003	0.00006
AFW1	0.008 ± 0.002	0.0016	0.00002 ± 0.000005	0.000005
AFW2	0.03 ± 0.01	0.0054	0.0001 ± 0.000006	0.000012
Algal medium	0.035 ± 0.014	0.003	0.00012 ± 0.000009	0.000003
Malt extraxt (ME)	0.4 ± 0.07	n.a.	0.03 ± 0.013	n.a.
YPD	2.0 ± 0.07	n.a.	2 ± 0.37	n.a.

M9 + 0.5%AA	1.5 ± 1.09	n.a.	0.35 ± 0.041	n.a.
M9	0.05	0.0018	n.a.	n.a.
LB	2.7 ± 0.17	n.a.	3.1 ± 0.21	n.a.
HMM + 0.5%AA	0.45 ± 0.2	n.a.	0.02 ± 0.0036	n.a.
HMM	0.015	0.0053	n.a.	n.a.
2% NaCl	0.012	0.0086	0.006 ± 0.00025	0.0043
0.9% NaCl	0.008 ± 0.001	0.006	0.0003 ± 0.00005	0.0046
0.9% NaCl + 0.01%AA	0.03 ± 0.02	n.a	0.015 ± 0.0036	n.a
0.9% NaCl + 0.05%AA	0.05 ± 0.01	n.a	0.05 ± 0.012	n.a
0.9% NaCl + 0.1%AA	0.14 ± 0.001	n.a	0.15 ± 0.066	n.a
0.9% NaCl + 0.5%AA	0.71 ± 0.3	n.a	0.6 ± 0.12	n.a

a chemical composition is shown in Table 1;

b Free Cu at Cu-ISE LOD in test media was calculated using the Visual MINTEQ chemical equilibrium model (see also Figure S3);

c 100% free ions were assumed in deionized water;

n.a.—not analysed.

**Table 3.** Limit of detection (LOD) of Cu-ISE (indicative of free Cu ions) and *Pseudomonas fluorescens* OS8::KnCueRPcopAlux Cu-biosensor (indicative of bioavailable Cu) for nano-CuO in selected ecotoxicological and micobiological media. Hydrodynamic diameter (D_h_) of the nano-CuO suspension in respective test medium is shown for comparison.

**Designation of the medium^a^**	**D_h_^b^±SD (Pdi)^b^**	**Cu-ISE_LOD_, mg/L**	**Cu-biosensor_LOD_, mg/L**
Deionized (DI) water	**195 ± 2 (0.2)**	**0.015 ± 0.003**	**n.a.**

Malt extract (ME)	391 ± 17 (0.2)	2	0.7 ± 0.2
YPD	1,644 ± 54 (0.4)	5.5	1.05 ± 0.2
M9 + 0.5%AA	525 ± 30 (0.2)	2.8	0.35 ± 0.04

LB	690 ± 21 (0.2)	1.4 ± 0.064	0.20 ± 0.06
HMM + 0.5%AA	786 ± 31 (0.2)	0.5	0.19 ± 0.05

0.9% NaCl	1,113 ± 31 (0.2)	0.009 ± 0.009	0.006 ± 0.0006
0.9% NaCl + 0.01%AA	952 ± 29 (0.2)	0.025 ± 0.0006	0.09 ± 0.025
0.9% NaCl + 0.05%AA	694 ± 33 (0.2)	0.03 ± 0.0005	0.085 ± 0.019
0.9% NaCl + 0.1%AA	504 ± 31 (0.2)	0.03 ± 0.0002	0.09 ± 0.013
0.9% NaCl + 0.5%AA	428 ± 35 (0.2)	0.05 ± 0.001	0.11 ± 0.03

a chemical composition of the media is presented in Table 1;

b Pdi—polydispersity index; measurement by Malvern Zetasizer Nano-ZS;

n.a.—not analysed.

**Table 4.** Toxicity (EC_50_ value) of CuSO_4_ and nano-CuO to different organisms in their conventional test or cultivation media. Free Cu at EC_50_-s was calculated according to Cu-ISE.

**Media**	**Test organism**	**EC_50_mg Cu/L**	**free Cu at EC_50_, mg Cu/L**
**Test compound: CuSO_4_**			

Osterhout’s medium	*Tetrahymena thermophila*	1.6 ^a^	1.1
AFW1	*Daphnia magna*	0.07 ^b^	0.49
AFW2	*Thamnocephalus platyurus*	0.044 ^b^	0.1
Algal medium	*Pseudokirchneriella subcapitata*	0.02 ^c^	0.15
Malt extract (ME)	*Saccharomyces cerevisiae*	11.4 ^d^	1.97
YPD	*Saccharomyces. cerevisiae*	368 ^e^	6.4
2% NaCl	*Vibrio fisheri*	0.64 ^f^	1.88
M9 + 0.5%AA	*Escherichia coli*	83.5 ^g^	0.97
0.9% NaCl	*Escherichia coli*	1.22 ^g^	2.66
0.9% NaCl + 0.1%AA	*Escherichia coli*	3.84 ^h^	0.19

**Test compound: nano-CuO**			

Malt extract (ME)	*Saccharomyces cerevisiae*	16.6 ^d^	11.1
0.9% NaCl + 0.1%AA	*Escherichia coli*	40.4 ^h^	90.2

a [19]; 24-h mortality test;

b [18]; 48-h mortality test;

c [46]; 72-h growth inhibition test;

d [54]; 8-h growth inhibition test;

e K. Kasemets, personal communication; 8-h growth inhibition test;

f [18]; 30-min bioluminescence inhibition test;

g [29]; 30-min bioluminescence inhibition assay with recombinant *E. coli*;

h [21]; 30-min bioluminescence inhibition test; AA—Cas-amino acids.
