# Supplementary material for: The Effect of Composition of Different Ecotoxicological Test Media on Free and Bioavailable Copper from CuSO4 and CuO Nanoparticles: Comparative Evidence from a Cu-Selective Electrode and a Cu-Biosensor
Source: Sensors (Basel). 2011 Nov 3;11(11):10502–21. doi: 10.3390/s111110502 (PMC3274297; doi:10.3390/s111110502)
Supplement: Supplementary file 1 [file sensors-11-10502-s001.pdf]

Supplementary Information

Aleksandr Käkinen [1,2,†], Olesja Bondarenko [1,3,†], Angela Ivask [1,*] and Anne Kahru [1,*]

[1] Laboratory of Molecular Genetics, National Institute of Chemical Physics and Biophysics, Akadeemia tee 23, Tallinn 12618, Estonia; E-Mails: aleksandr.kakinen@kbfi.ee (A.K.); olesja.bondarenko@kbfi.ee (O.B.)

[2] Department of Chemical and Materials Technology, Tallinn University of Technology, Ehitajate tee 5, Tallinn 19086, Estonia

[3] Department of Gene Technology, Tallinn University of Technology, Ehitajate tee 5, Tallinn 19086, Estonia

* Authors to whom correspondence should be addressed; E-Mails: angela.ivask@kbfi.ee (A.I.); anne.kahru@kbfi.ee (A.K.); Tel.: +372-6-398-382 (A.I.); +372-6-398-373 (A.K.); Fax: +372-6-398-382 (A.I.); +372-6-398-382 (A.K.).

[†] These authors equally contributed to this work.

Received: 20 September 2011; in revised form: 20 October 2011 / Accepted: 31 October 2011 / Published: 3 November 2011

Figure S1. Example for the calculation of limit of detection (LOD) of Cu-ion selective electrode, as recommended for ion-selective electrodes by IUPAC [1]. Log(10) of the added Cu was plotted against the electrode potential and the crossing point between the linear segment of the electrode potential (diagonal dotted grey line) and the line representing background potential of the electrode (horizontal dotted grey line) was seeked. The concentration of added Cu at which the two lines were crossing (vertical dotted grey line) was designated as the limit of detection (Cu-ISE$_{LOD}$). In the current example, LOD of Cu-ISE is 0.021 mg Cu/L = 3.2×10^{-7} M. *Prior* measurement, the ionic strength of all solutions was adjusted by adding 5 M $NaNO_3$ in a ratio 1:50 ($NaNO_3$:sample).

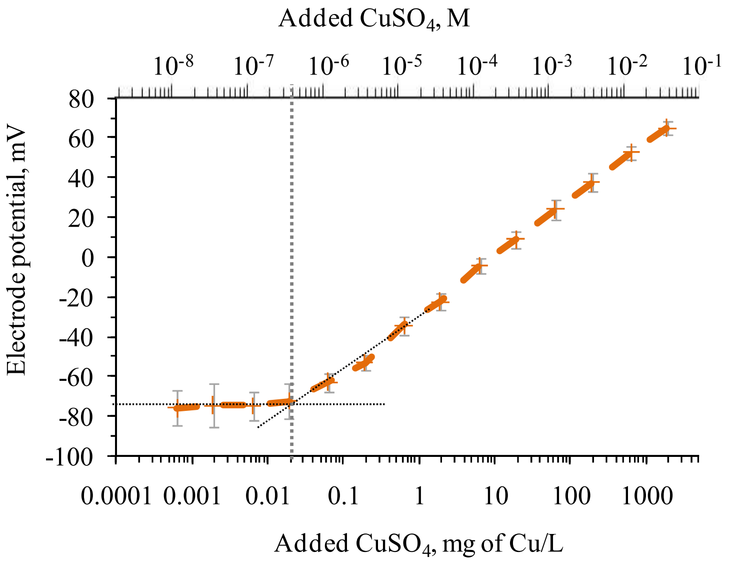

Figure S2. Example for the calculation of free Cu at a certain $CuSO_4$ concentration in a specific media (Malt extract (ME) as an example). Normalized electrode potentials for DI water (100% of added Cu assumed in free form) and for the given medium (ME) as a function of added Cu are plotted. Then, the electrode potential at desired concentration (11.4 mg of added Cu/L in this case) in the medium is read (1) and the Cu concentration corresponding to that electrode potential in DI water is found (2). This Cu concentration in DI water (1.97 mg/L in this case) is considered as the free Cu concentration in this media at the given (11.4 mg of added Cu/L) concentration.

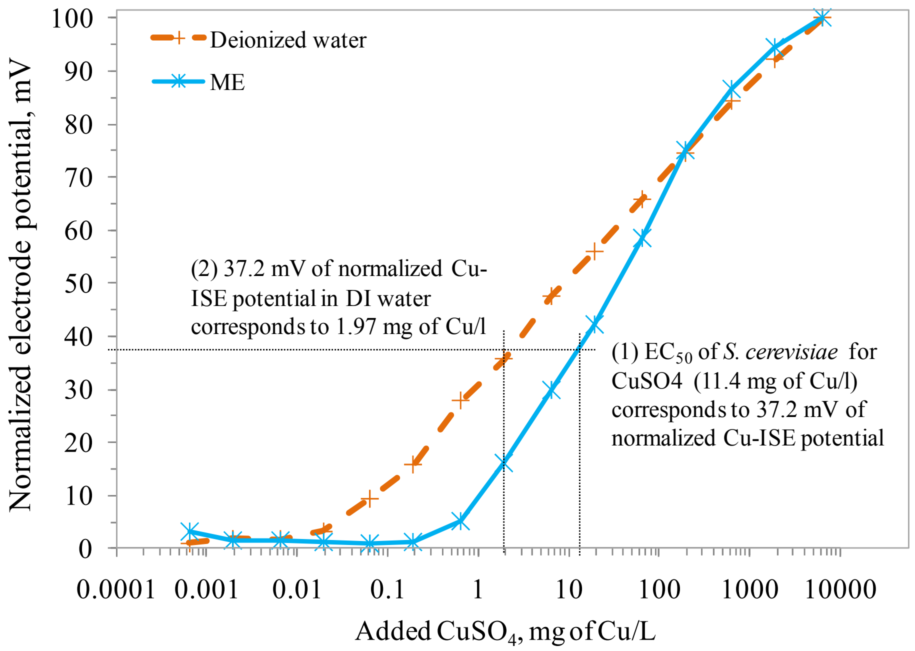

Figure S3. Example for the calculation of limit of detection (LOD) of Cu-biosensor bacteria *Pseudomonas fluorescens* OS8::KnCueRPcopAlux in HMM media supplemented with 0.5% Cas-aminoacids (Table 1). (**a**) representation of fold induction of Cu sensor bacteria with increasing Cu concentrations (dose-response curve); in our earlier studies [2,3], concentration of added metals resulting in 2-fold induction of a bacterial sensor (marked with dashed lines) was considered significant induction of the sensor over the background signal and thus, suggested as LOD. (**b**) representation of the same data as % induction of the bacterial sensor; in this plot, concentration of added metals resulting in 20% induction of the bacterial sensor of maximal induction potential of the sensor in the current test conditions (marked with dashed lines) was considered as LOD. This normalization was performed because the fold induction of the bacterial sensor varied along with the nutrient profile in different media studied. For panel (a) the LOD is 0.13 mg/L; for panel (b) the LOD is 0.2 mg/L.

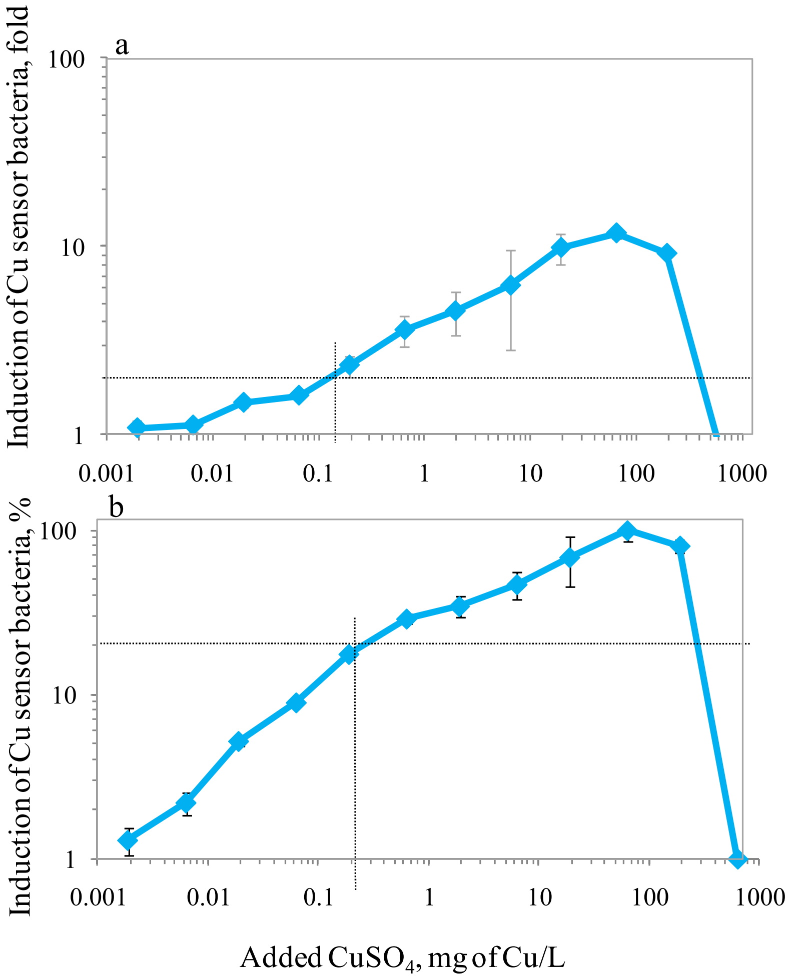

Figure S4. Speciation of Cu (added to respective media as $CuSO_4$) in different test media containing mainly mineral salts or very low levels of organic components (glucose and β-glycerophosphate). Only main species (constituting at least 5% of the total Cu species over the concentration range tested) are indicated. AFW1—Artificial freshwater 1; AFW2—Artificial freshwater 2; HMM—Heavy metal MOPS medium, M9 (see the content of these media in Table 1). Chemical equilibrium model Visual MINTEQ 2.51 [4] was used for the calculations.

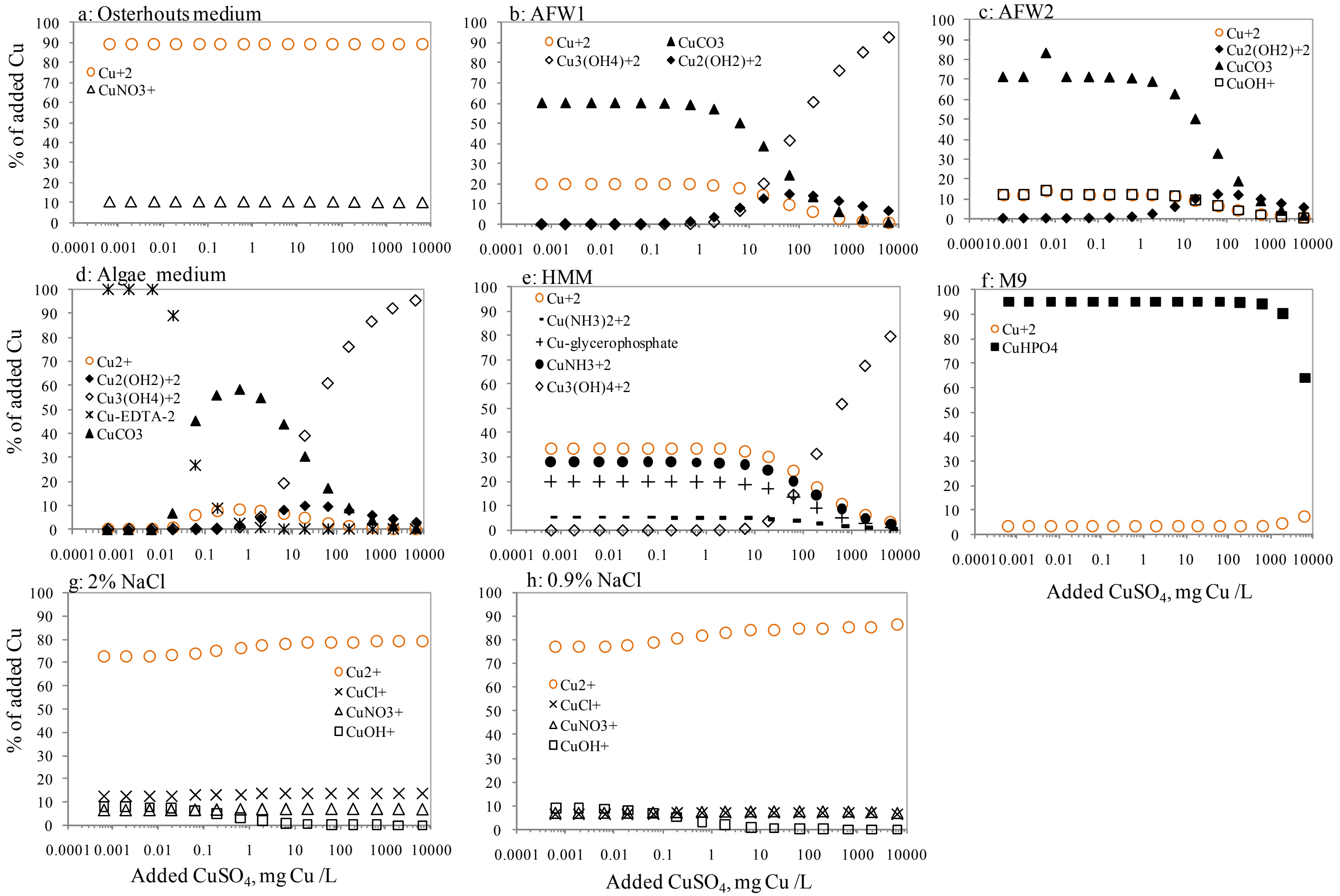

References

1. Buck, P.B.; Lindner, E. Recommendations for nomenclature of ion-selective electrodes. *Pure Appl. Chem.* **1994**, *66*, 2527-2536.
2. Hakkila, K.; Green, T.; Leskinen, P.; Ivask, A.; Marks, R.;Virta, M. Detection of bioavailable heavy metals in EILATox-oregon samples using whole-cell luminescent bacterial sensors in suspension or immobilized onto fibre-optic tips. *J. Appl. Toxicol.* **2004**, *24*, 333-342.
3. Ivask, A.; Rolova, T.; Kahru, A. A suite of recombinant luminescent bacterial strains for the quantification of bioavailable heavy metals and toxicity testing. *BMC Biotechnol.* **2009**, *9*, 41.
4. Gustafsson, J. *Visual Minteq v2.51*; The Royal Institute of Technology (KTH): Stockholm, Sweden, 2008.

© 2011 by the authors; licensee MDPI, Basel, Switzerland. This article is an open access article distributed under the terms and conditions of the Creative Commons Attribution license (http://creativecommons.org/licenses/by/3.0/).
